# Transcriptional activation of p21^Waf1^ contributes to suppression of HR by p53 in response to replication arrest induced by camptothecin

**DOI:** 10.18632/oncotarget.25172

**Published:** 2018-05-22

**Authors:** Larisa Y. Romanova, Frederick Mushinski, Alexander L. Kovalchuk

**Affiliations:** ^1^ The Laboratory of Cancer Biology and Genetics, Center for Cancer Research, National Cancer Institute, NIH, Bethesda, Maryland, USA; ^2^ The Virology and Cellular Immunology Section, Laboratory of Immunogenetics, National Institute of Allergy and Infectious Diseases, NIH, Rockville, Maryland, USA

**Keywords:** RPA phosphorylation, p53, transcriptional activation, homologous recombination

## Abstract

The inhibitory effect of p53 on homologous recombination (HR) is exerted through sequestration of replication protein A (RPA). Release of the p53/RPA complex in response to replication stress is crucially dependent on the phosphorylation status of both proteins and is required for efficient DNA repair by HR. Phosphorylation of RPA within its RPA2 subunit by cyclin-dependent kinases (CDK) is an early event in the replication stress response. Here we investigated the role of transcriptional activation of the p53 downstream target, p21^Waf1^, on RPA2 phosphorylation, the stability of the p53/RPA complex and HR in cells undergoing replication arrest induced by camptothecin (CPT). We show that in CPT-treated cells, activation of p53 and p21^Waf1^ impedes RPA2 phosphorylation, while their depletion by siRNA stimulates it. The p53/RPA complex is more stable in wild-type cells than in cells depleted of p21^Waf1^. We used nocodazole-synchronized cells treated with CPT at the entrance to S phase to assess rates of HR. Regardless of their p53 or p21^Waf1^ status, the cells proceed through S phase at a similar rate and enter G2. While HR is low in wild-type cells and high in p53-depleted cells, only partial inhibition of HR is observed in the p21^Waf1^-depleted cells. This correlates with the extent of RPA sequestration by p53. Thus, in CPT-treated cells, p53-induced transcriptional activation of p21^Waf1^ regulates RPA2 phosphorylation, the stability of the p53/RPA complex and HR.

## INTRODUCTION

Precise genome duplication is, by its nature, a very complex and challenging process. It requires the action of many molecular players that need to exert their functions in a perfectly concerted manner. For DNA replication to be accurately completed, the replication fork must frequently overcome a multitude of structurally unrelated obstacles such as DNA lesions, transcribing RNA polymerases, and tightly bound protein–DNA complexes. Stalling of the replication machinery during S phase creates a hazardous situation for cells. This often results in formation of double strand breaks and replication fork collapse thus contributing to genomic instability and tumorigenesis [1–3].

Camptothecin (CPT) and its derivatives used in anticancer therapy are highly selective inhibitors of topoisomerase I (Top I) that irreversibly lock the enzyme on DNA during the intermediate step of enzymatic cleavage (reviewed in [4]). In proliferating cells, a collision of replication machinery with these complexes generates double-strand breaks. In contrast to the double-strand breaks produced by ionizing radiation or Sce-I digestion, these are one-ended, making them a poor substrate for non-homologous end joining (NHEJ). Such lesions are therefore repaired primarily by HR.

The outcome of CPT therapy is known to depend on p53 status, with p53 or p21^Waf1^ mutant cells being more sensitive to therapy than p53-proficient ones. Differential sensitivity to CTP therapy is attributed to the ability of p53-proficient cells to undergo an extended G2 arrest providing cells additional time for DNA repair. Lack of G2 arrest and high rates of HR were suggested to contribute to drug resistance and clonal expansion of CTP-treated p53-negative cells [5].

Human RPA, consisting of ∼70-kDa (RPA1), 30-kDa (RPA2), and 14-kDa (RPA3) subunits undergoes extensive phosphorylation by several kinases on its middle subunit, RPA2 [6]. The N-terminal Ser^23^ and Ser^29^ of RPA2 undergo cell cycle- and genotoxic stress-dependent phosphorylation by cyclin-CDK complexes [7–11]. Phosphorylation of these residues in bleomycin- or CPT-treated cells precedes and is required for RPA2 modifications by other kinases [12]. Persistent RPA-ssDNA intermediates formed as a result of replication arrest recruit the ATR-ATRIP complex leading to the ATR-dependent phosphorylation of RPA2 at Ser^33^ [13], and activation of CHK1, an S phase checkpoint regulator [14, 15]. Collapse of replication forks and formation of double-strand breaks stimulate ATM and DNA-PK kinases that phosphorylate RPA2 at Thr^21^ and induce CHK2, another S phase checkpoint regulator [15, 16]. The remaining sites, Ser^4^, Ser^8^, Ser^11^, Ser^12^, and Ser^13^ are phosphorylated in response to genotoxic stress, presumably by DNA-PK.

p53 is known to have pleiotropic functions in DNA repair, replication and recombination [17–19]. p53 suppresses spontaneous inter- and intra-molecular HR thus facilitating genomic integrity [18, 20, 21]. A number of findings suggest that p53 may be directly involved in recombination control. It binds to recombination intermediates and Holliday junctions *in vitro* and this is required for efficient inhibition of HR *in vivo* [22, 18] [23, 24]. In addition, p53 interacts with various proteins involved in HR, including the Rad51 recombinase, and the largest subunit of RPA - RPA1 [25–27]. We earlier reported that disruption of a RPA binding site within p53 by mutations of Trp-53 and Phe-54 leads to upregulation of spontaneous and DNA replication stress-induced HR [28, 29]. Dissolution of the p53/RPA complex with a subsequent upregulation of HR occurs *in vivo* in response to replication arrest induced by UV or CPT [30, 31].

NMR spectroscopy analyses suggested that phosphorylated forms of RPA2 generated during DNA damage compete with p53 for RPA1 binding [32] destabilizing the RPA/p53 complex. Specifically, phosphorylation of RPA2 at Ser^4/8^ by DNA-PK in response to CPT is required for both RPA release from p53 and efficient DNA repair by HR [31]. These, and other experiments showing the inhibitory effect of the transactivation-deficient p53(22.23) mutant on HR contributed to the notion of transactivation-independent regulation of HR by p53.

Here we report that the level of RPA2 phosphorylation in CPT-treated cells could be regulated by p53. In such cells, p53 impedes hyperphosphorylation of RPA2 in a manner that requires p21^Waf1^ transcriptional activation, thus contributing to stabilization of the p53/RPA complex. In contrast, depletion of p53 or p21^Waf1^ by siRNA leads to RPA hyperphosphorylation and RPA release from p53. As expected, HR rates in these cells correlate with the pattern of RPA sequestration by p53.

## RESULTS

### Phosphorylation of RPA in cells treated with CPT contributes to dissociation of the RPA/p53 complex

**Figure 1:**
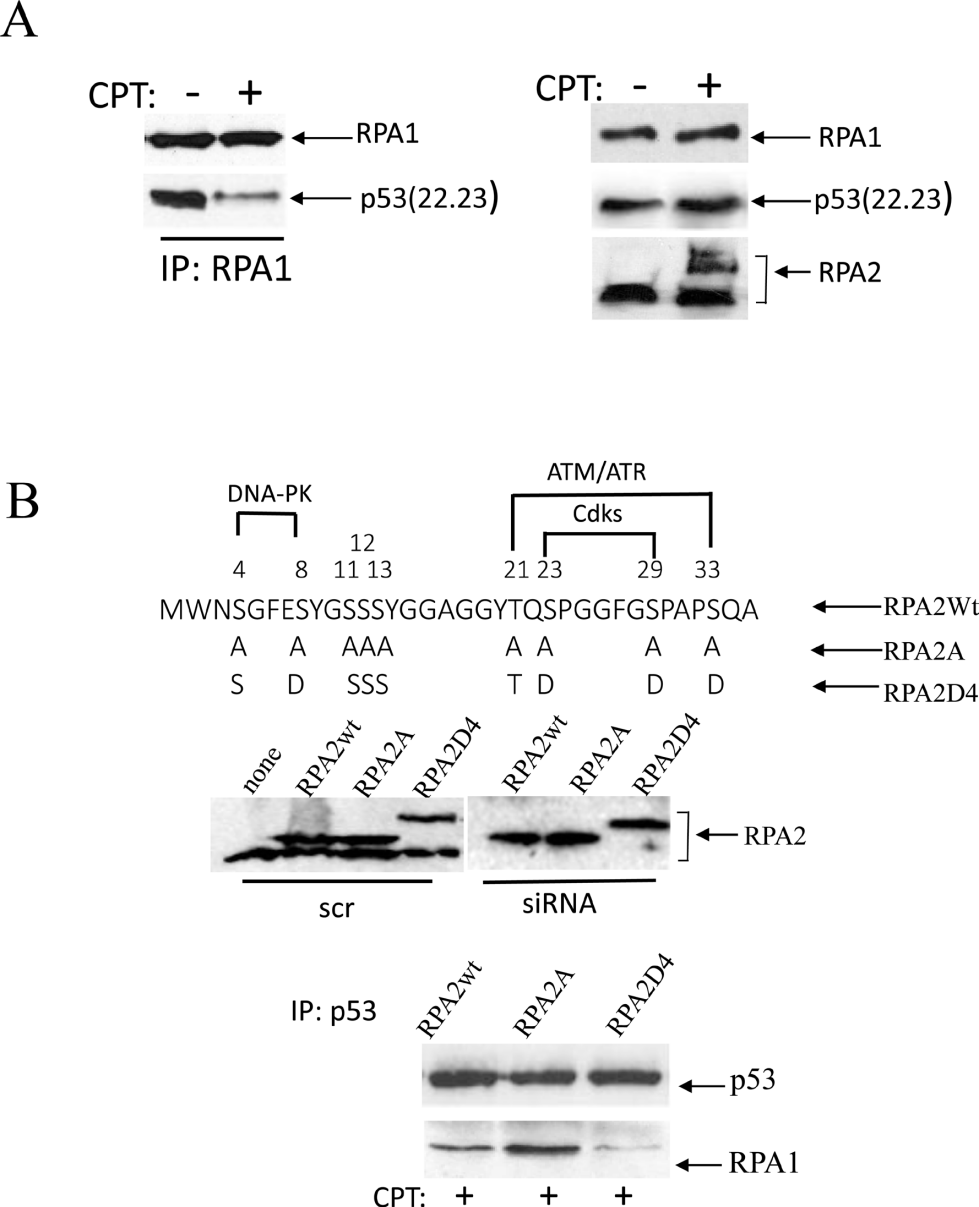
Phosphorylation of RPA in response to replication arrest induced by CPT contributes to the dissociation of the RPA/p53 complex (**A**) Cells were collected for analysis following one-hour treatment with 500 nM CPT. p53-negative H1299 cells were transfected with the transactivation-deficient p53(22.23) mutant. p53 binding was assessed following RPA1 immunoprecipitation. The expression levels of p53(22.23), RPA1 and phosphorylation of RPA2 in untreated cells were used as loading controls. (**B**) The residues within the N-terminal RPA2 domain reported to be phosphorylated by CDKs, ATR/ATM and DNA-PK were replaced with alanine or glutamic acid, thus producing RPA2A or RPA2D4 mutants that imitate non-phosphorylated or phosphorylated forms of RPA2, respectively. The consensus sites for the kinases are indicated. Expression levels of the recombinant wild type RPA2 and the mutants in A549 cells prior to or after siRNA silencing of endogenous RPA2. The cells with the silenced endogenous RPA2 were treated with 500 nM CPT for one hour. After p53 immunoprecipitation, RPA binding was analyzed on western blots with anti-RPA1 antibody.

We earlier reported that the p53(22.23) transactivation-deficient mutant efficiently binds RPA1 [28]. We investigated the effect of CPT on RPA2 phosphorylation and the stability of RPA complex with recombinant p53(22.23). p53-negative H1299 cells were transfected with the transactivation-deficient p53(22.23) mutant, and p53 binding to RPA1 in response to CPT treatment was assessed following RPA1 immunoprecipitation (Figure 1A). CPT leads to an apparent loss of p53(22.23) from immunoprecipitated RPA1. Western blotting shows some accumulation of the mutant p53(22.23) and robust phosphorylation of RPA2. This suggests that RPA2 phosphorylation contributes to dissolution of the RPA/p53 complex.

To investigate the role of RPA2 phosphorylation on the stability of the complex with endogenous p53, we replaced the endogenous RPA2 subunit in A549 cells with the Myc-tagged recombinant constructs: wild-type, phosphorylation-deficient or phosphorylation-mimetic RPA2 mutants (Figure 1B). In the latter two constructs, serines or threonines within the putative phosphorylation sites of CDKs, ATR/ATM or DNA-PK were replaced with alanine or glutamic acid, respectively. A replacement strategy of the endogenous RPA2 with the recombinant RPA2 constructs was described earlier [12, 31, 33, 34]. Expression levels of the recombinant RPA2 prior or following silencing of the endogenous RPA2 is shown on Figure 1B. Following addition of CPT, equal amounts of p53 were immunoprecipitated from each derivative cell line. Compared to wild-type RPA2, the phosphorylation-deficient RPA2 mutant stabilizes the RPA1/p53 complex and the phosphorylation-mimetic RPA2 contributes to its dissociation. This confirms that in CPT-treated cells RPA phosphorylation disrupts binding of RPA and p53.

### Effect of p53 and p21^waf1^ depletion on RPA2 phosphorylation and stability of the p53 complex with RPA

**Figure 2:**
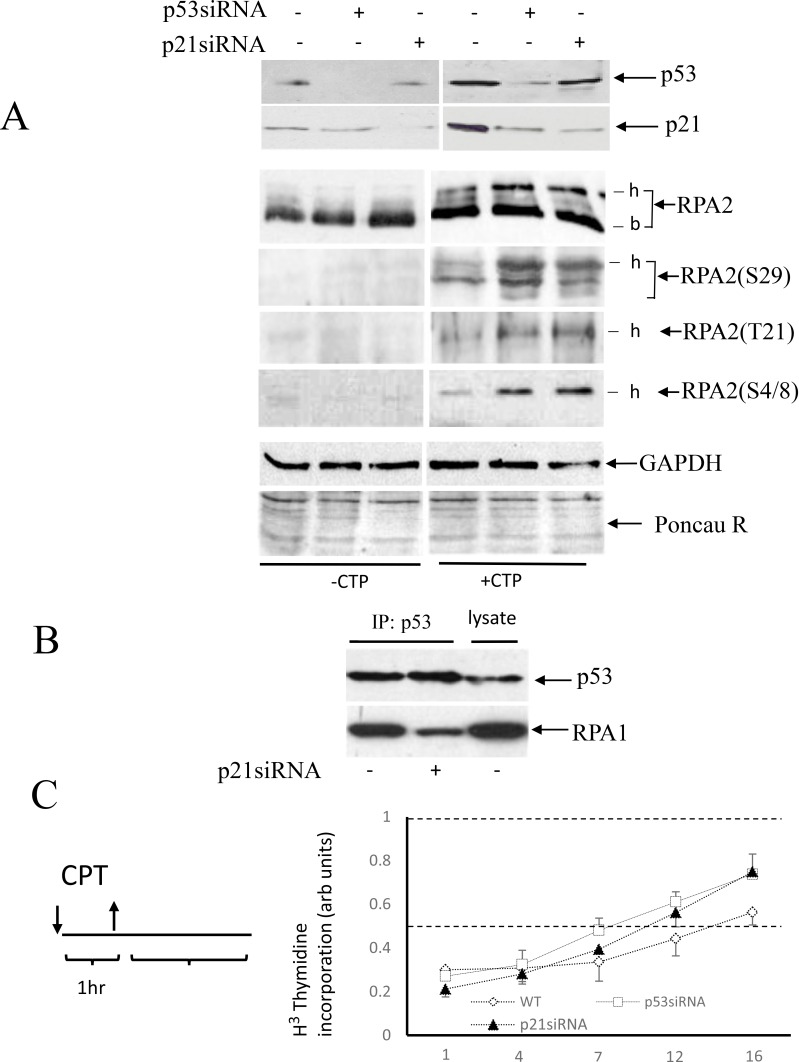
Effect of p53 or p21^Waf1^ siRNA depletion on RPA2 phosphorylation and stability of p53 complex with RPA (**A**) A549 cells were collected following one-hour 500 nM CPT treatment and the levels of p53 and p21^Waf1^ as well as an overall RPA2 phosphorylation or phosphorylation at the residues Ser^29^, Tyr^21^ and Ser^4/8^ were analyzed by western blots; -b and -h indicate base non-phosphorylated and hyper-phosphorylated RPA2 forms. GAPDH and Ponceau Red staining were used as protein loading controls. (**B**) In CPT-treated cells, p21^Waf1^ was silenced by siRNA as indicated. Stability of the RPA/p53 complex was analyzed by western blots following p53 or RPA1 immunoprecipitation. 15% of the total cell lysate was loaded in the last lane. (**C**) A scheme of cell treatment with CPT used in our experiments. Cells were pulse-treated with 500 nM CPT for one hour and later maintained in a drug-free medium. DNA synthesis in intact A549 cells or its p53- or p21^Waf1^ siRNA depleted derivatives was analyzed by the [^3^H]-thymidine incorporation assay at different time intervals following CPT pulse treatment. The results are normalized to the DNA synthesis rate of untreated cells.

RPA2 phosphorylation by CDKs is an early event in cell replication arrest. This is followed by phosphorylation by other kinases - ATM, ATR, and DNA-PK. Because p21^Waf1^ is a potent inhibitor of CDKs, we investigated the effect of p53/p21^Waf1^ activation on RPA2 phosphorylation in response to CPT. In A549 cells, endogenous p53 or p21^Waf^ was depleted with siRNA. A brief, one-hour treatment with CPT had no noticeable effect on cell cycle distribution (data not shown). In untreated cells, RPA2 is not phosphorylated and the levels of p53 and p21^Waf1^ are low (Figure 2A). CPT treatment leads to an increase in overall phosphorylation of RPA2, and siRNA depletion of p53 or p21^Waf1^ further facilitates RPA2 phosphorylation. Phosphorylation of Ser^29^, Thr^21^, and Ser^4/8^ follows similar patterns (Figure 2A). Thus, activation of the p53/p21^Waf1^ axis delays RPA2 phosphorylation.

Because RPA2 phosphorylation regulates the stability of RPA/53 complex, we investigated the effect of p21^Waf1^ siRNA depletion on stability of the p53/RPA complex. Equal amounts of p53 were immunoprecipitated from CPT-treated parental or p21^Waf1^-depleted cells. In this experiment, siRNA inhibition of p21^Waf1^ contributed to dissociation of the p53/RPA complex (Figure 2B). Thus, in CPT-treated wild-type, p21^Waf1^- and p53-depleted cells, RPA is progressively released from p53. These data show that RPA2 phosphorylation regulated by the p53/p21^Waf1^ axis affects the stability of the p53/RPA complex.

In the experiments that require an extended time for evaluation, one-hour pulse treatment with 500 nM CPT was followed by maintaining cells in drug-free medium. As evidenced by [^3^H]-thymidine incorporation assay, pulse-treatment with CPT elicits a robust inhibition of DNA replication in all three lines that lasts up to 7 hours and is followed by gradual restoration of DNA synthesis. Within 16 hours, wild-type and p53- or p21^Waf1^-compromised cells restore DNA synthesis to 55% and 75% of untreated control level, respectively. This difference is attributed to the ability of the p53-positive cells to undergo G2/M arrest following CPT treatment.

### Effect of p53 and p21^Waf1^ depletion on rates of HR

**Figure 3:**
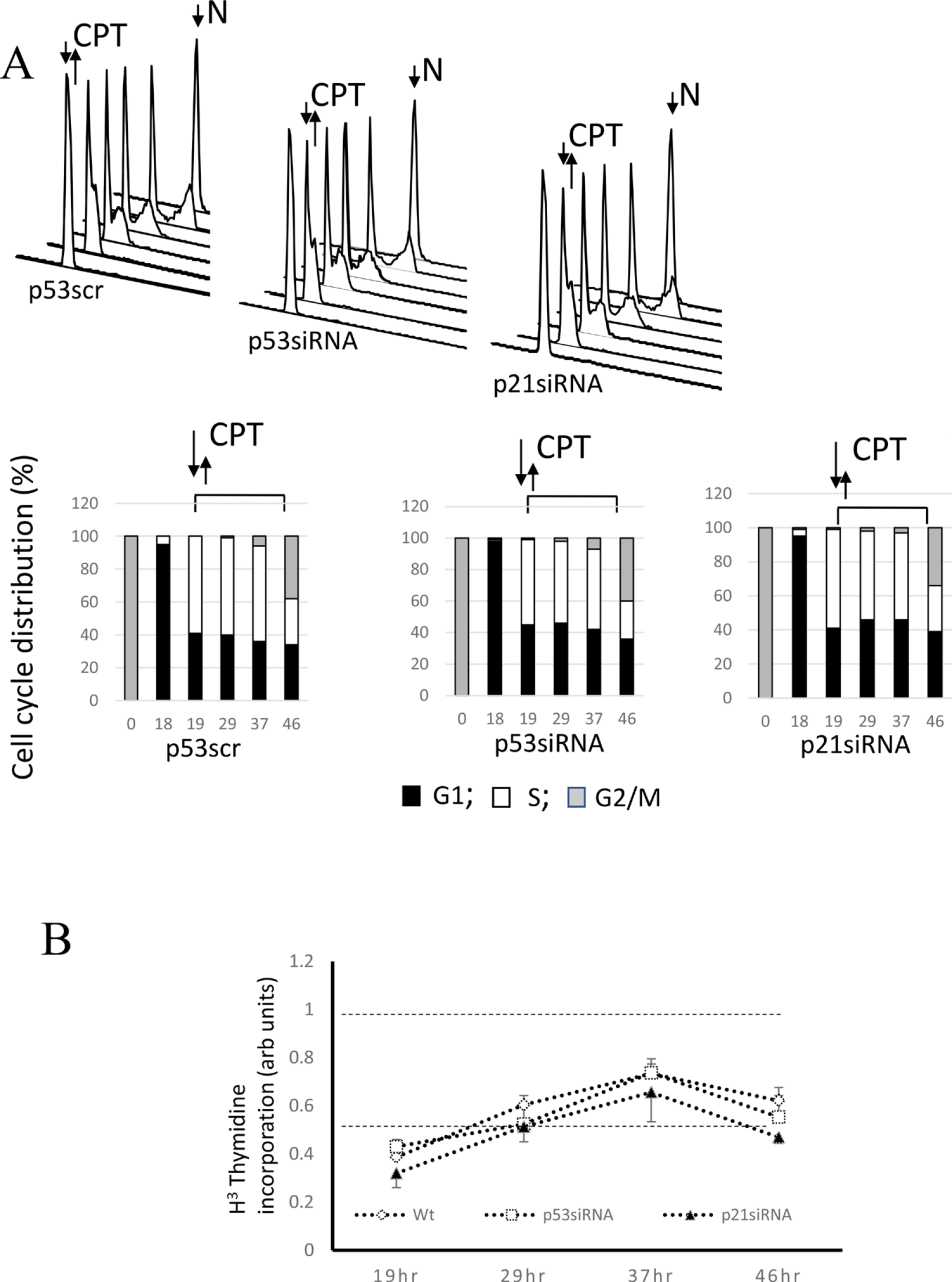

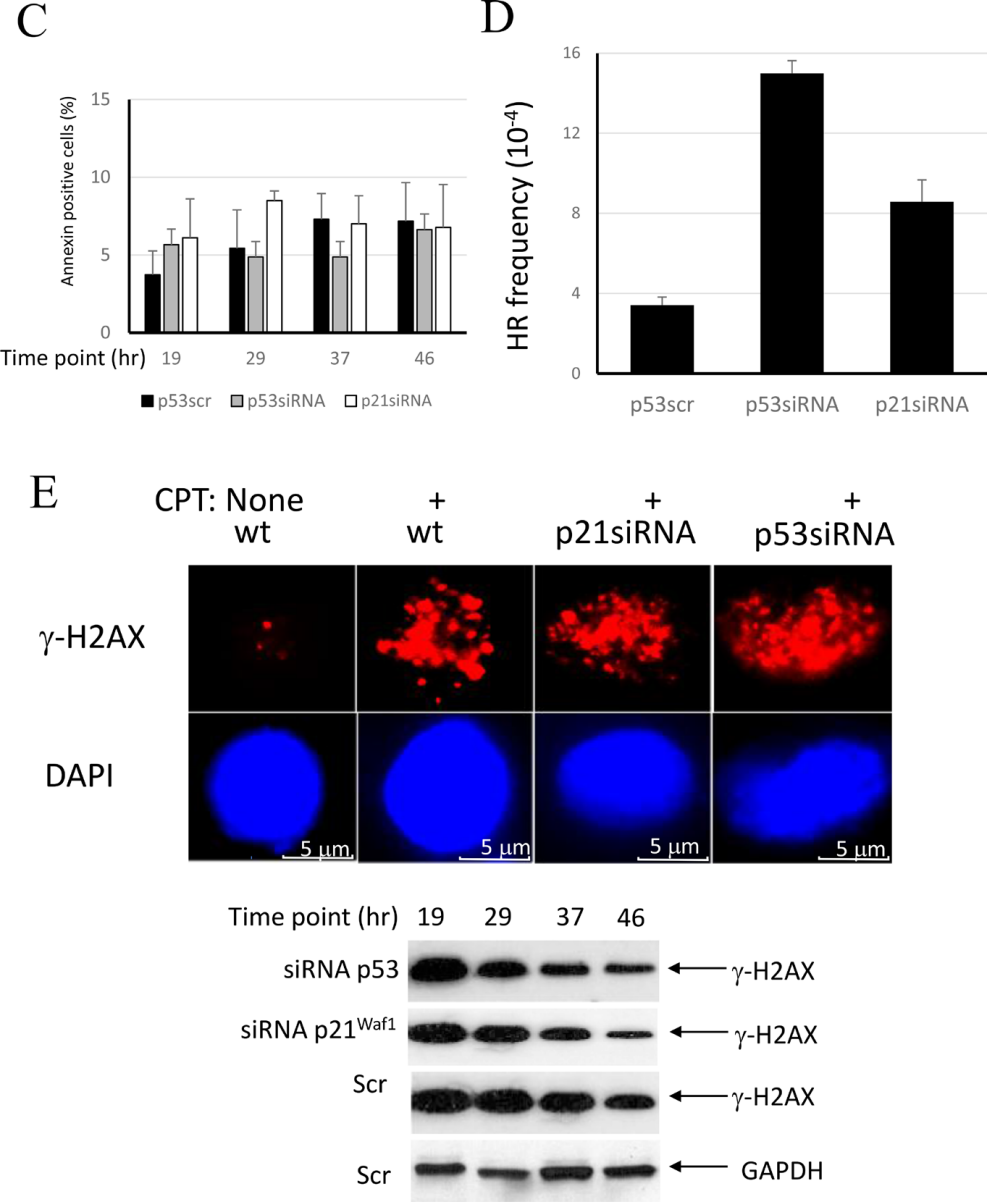
Involvement of the p53/p21^Waf1^ axis in regulation of HR in response to replication arrest by CPT (**A**) Control A549 cells or cells with p53 or p21^Waf1^ silenced by siRNA were synchronized in mitosis by nocodazole. After release from nocodazole arrest, cells were allowed to progress through the cell cycle and collected at different time-points to evaluate cell cycle progression by flow cytometry. Upon entrance into S phase, the cells were pulse-treated with CPT and later maintained in a drug-free medium. Cell cycle profiles and derivative bar graphs show the relative frequencies of cells in each stage of cell cycle at the indicated time points. (**B**) DNA synthesis was assessed at different time intervals following CPT treatment by [^3^H]-thymidine incorporation assay and normalized to the DNA synthesis rate of untreated cells. (**C**) The percentages of apoptotic cells at the indicated times were assessed by flow cytometry following staining with FITC-conjugated Annexin V. (**D**) The cell line A549 carries a stably-transfected recombinant reporter construct, pDR-GFP [49]. The cells were harvested 27 hours following CPT addition and the HR frequencies were analyzed. Chi-square tests detected significant differences between p21siRNA and p53siRNA depleted cells (χ^2^ = 26.3; *p* < 0.0001), between p21siRNA-depleted cells and control (χ^2^ = 30.0; *p* < 0.0001) and between cells depleted of p53 by siRNA and controls (χ^2^ = 104.0; *p* < 0.0001). (**E**) Double stand breaks in the nuclei without or immediately following pulse CPT treatment were assessed by immunofluorescence using anti-γ-H2AX antibodies. Nuclei were counterstained with DAPI. Persistence of double strand breaks was analyzed by Western Blot using anti-γ-H2AX antibodies at different time points following CPT removal. GAPDH expression was used as a control.

It was suggested that accumulation of p53-proficient cells in G1 in response to genotoxic stress contributes to inhibition of HR by the tumor suppressor, p53 [43]. To dissociate the effect of p53 on HR from its cell cycle effect, we used nocodazole-synchronized cells pulse-treated with CPT at the entrance to S phase. The doubling time of A549 cells in our hands is 38 hours with S phase lasting approximately 16 hours (unpublished data), a time slightly shorter than that reported previously [35]. A significant delay of S phase duration (up to 27 hours) was observed following pulse-treatment with CPT (Figure 3A), with all derivatives, wild-type, p53 - or p21^Waf1^-depleted cells proceeding through S phase with a similar rate and entering G2 (Figure 3A).

Consistently, the experiments examining [^3^H]-thymidine incorporation show that the parental cells and both derivative cell lines progressively restore DNA synthesis following an initial robust inhibition (Figure 3B). There is no significant difference in the kinetics of DNA synthesis restoration between these cell lines, confirming the results of cell cycle experiment. The independence of S phase progression from p53 status following CPT pulse treatment is in line with prior findings [36]. The pattern of DNA synthesis restoration is different in unsynchronized cell cultures (Figure 2C), probably because CPT-treated the p53-positive cells, but not p53- or p21^Waf1^ -depleted cells undergo an extended senescence-like G2 cell cycle arrest.

We next assessed viability of the cell lines at different times during the experiment (Figure 3C). Approximately 4–7% of cells were annexin V positive. There was no time- or cell line-related differences in staining pattern, thereby suggesting that cell viability does not change over the course of the experiment.

The use of SCE-I-inducible pDR-GFP recombination substrates for studies of one-ended double-strand break repair resulted from replication arrest by hydroxyurea, thymidine or camptothecin was described earlier [34, 37–41]. Compared to intact parental cells, cells depleted of p53 show over a 3-fold upregulation of HR, while p21^Waf1^-depleted cells demonstrate only a 2-fold increase in rates of HR (Figure 3D). Chi-square analysis shows statistically significant differences between HR frequencies of all three lines (Figure 3D legend). RPA sequestering by p53 was shown to be responsible for the inhibition of HR [28, 29, 31]. The extent of HR inhibition by p53 (Figure 3D) parallels the pattern of RPA sequestering, with maximal, partial and no sequestration in wild-type, p21^Waf1^- or p53-negative cells, respectively (Figure 2).

Immunofluorescent staining for phosphorylated H2AX (γ-H2AX) showed that CPT treatment induced double strand breaks in all cell lines. Immunoblotting for γ-H2AX demonstrated a gradual decrease of staining over the course of the experiment with the pattern reflecting the recombination activity. GAPDH immunostaining did not follow this trend. Thus, our data suggest that p53- and 21^waf1^-deficient cells repair double-strand breaks more efficiently than the parental cells (Figure 3E).

Our results suggest that a transactivation-independent mechanism of HR regulation by p53 is complemented by a transactivation-dependent mechanism. This is evidenced from only partial ability of p21^Waf1^-depleted cells to inhibit HR.

### Involvement of p53 and p21^Waf1^ in the regulation of RPA2 phosphorylation and homologous recombination in other cell line models

**Figure 4:**
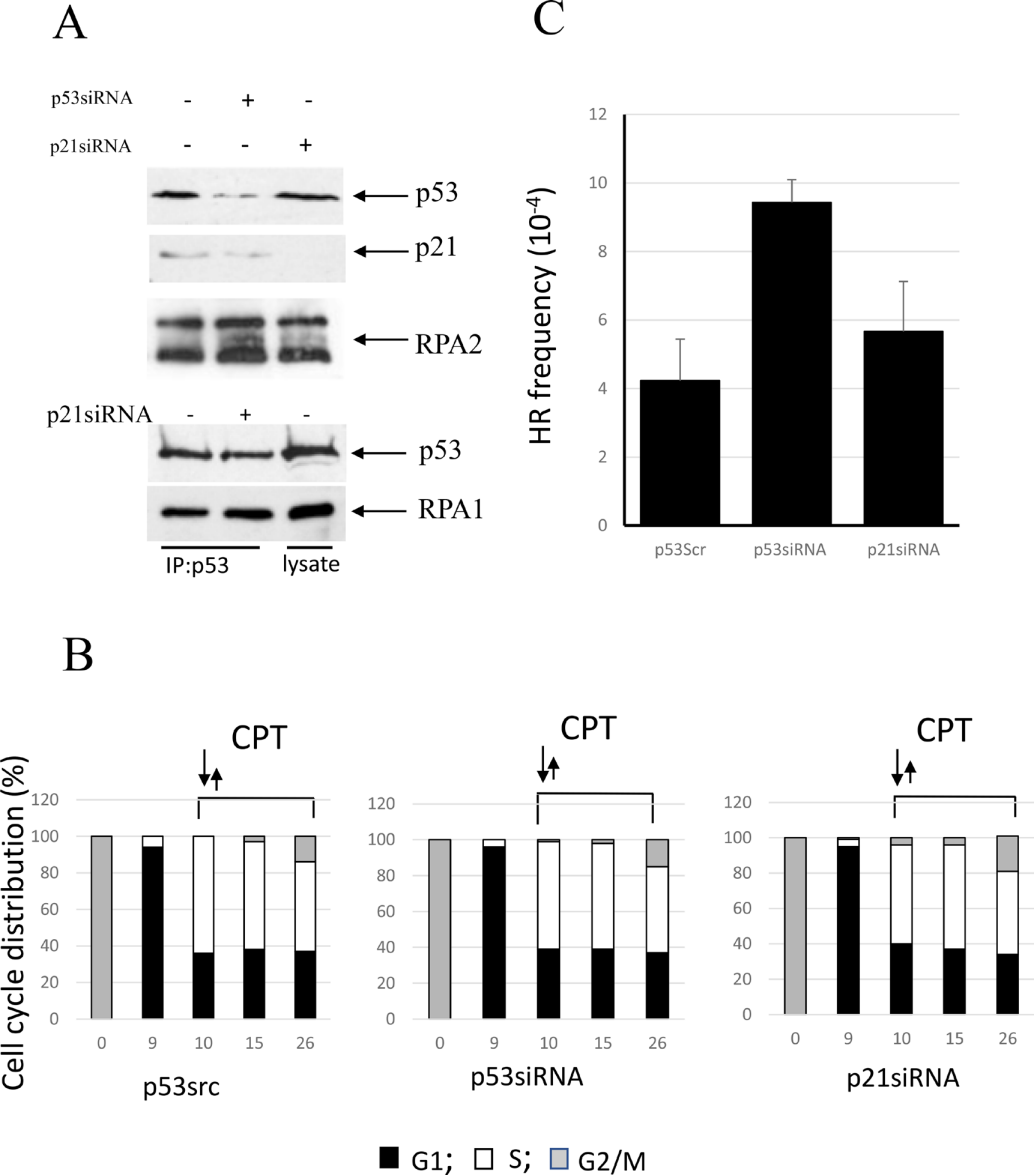
Involvement of p53 in the regulation of CPT-induced RPA2 phosphorylation and homologous recombination in U2OS cells (**A**) Following 1 hour 500 nM CPT treatment, U2OS and its p53- or p21^waf1^-depleted derivatives were harvested. Overall RPA2 phosphorylation, p53 and p21^waf1^ expression levels were assessed by western blotting. Binding of p53 to RPA was analyzed by western blotting following RPA1 immunoprecipitation. (**B**) U2OS cells were synchronized, pulse-treated with CPT upon entry into S phase and further maintained in drug-free medium. The bars represent the relative cell frequencies at different stages of the cell cycle at the indicated times. (**C)** HR frequency within pDR-GFP recombination substrate was measured 16 hours following CPT treatment.

In U2OS cells, an upregulation of p53 in response to genotoxic stress is accompanied by attenuated accumulation of p21^Waf1^ [42]. We show here that CPT pulse-treatment of U2OS cells leads to accumulation of p53, but not p21^Waf1^ (Figure 4A). Consistently, depletion of p21^Waf1^ with siRNA does not affect the rate of RPA2 phosphorylation and the stability of the p53/RPA complex. U2OS cells were synchronized by nocodazole and later pulse-treated with CPT at the entrance to S phase (Figure 4B). After CPT removal, a progression of the cells through S phase is not affected by p53 or p21^Waf1^ status. As expected, p53 depletion by siRNA leads to upregulation of HR, while its functional depletion by siRNA silencing of p21^Waf1^ does not (Figure 4C).

**Figure 5:**
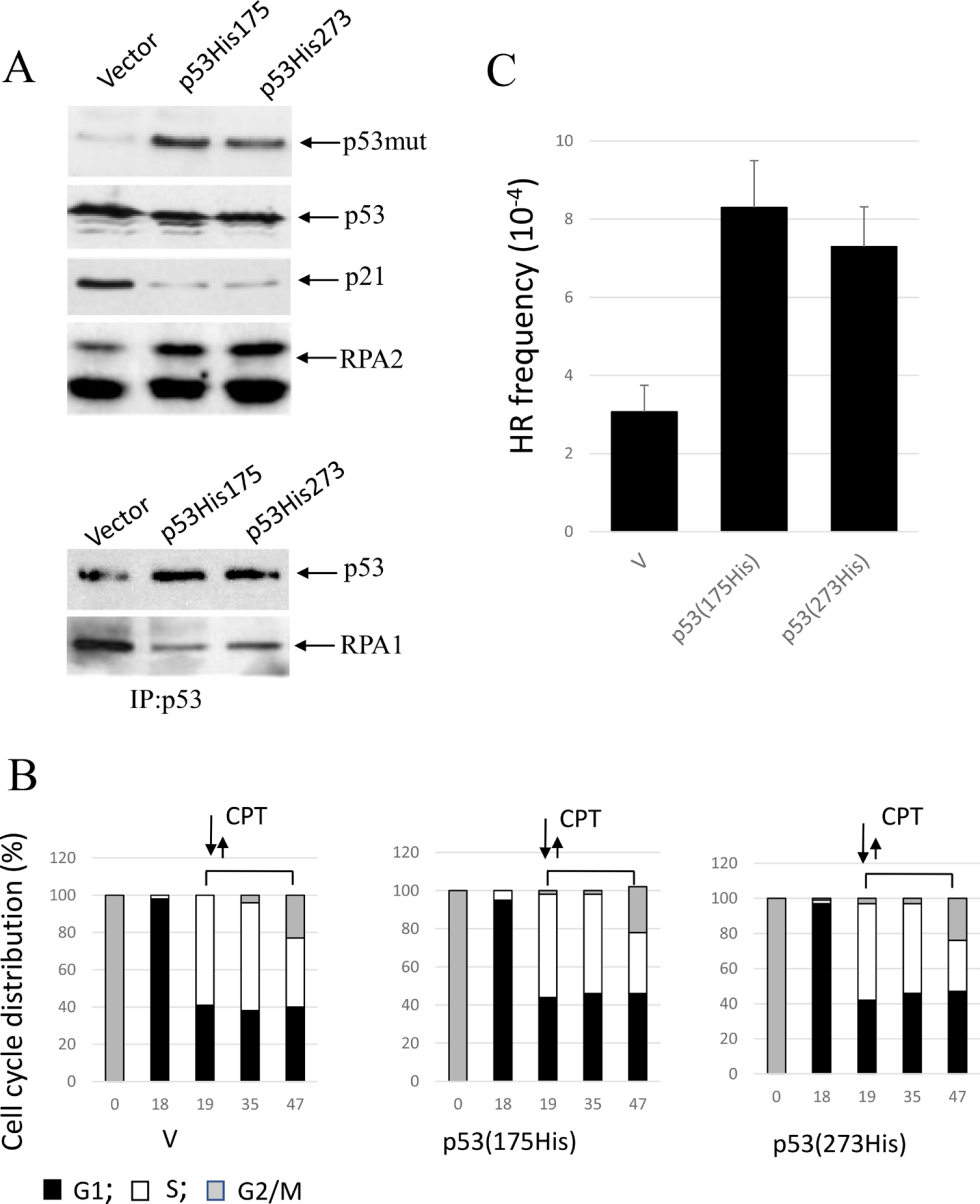
Expression of conformational or DNA-binding p53 mutants, p53(His175) and p53 (His273), in A549 cells affects CPT-induced RPA2 phosphorylation and HR (**A**) Cells were harvested following one-hour CPT treatment at 500 nM. Expression of p53, p21^Waf1^ and an overall RPA2 phosphorylation were analyzed by western blot with antibodies specific for p21^Waf1^, RPA1, mutant p53 (ab32049, Abcam) or for both wild-type and the mutant p53 (Pab240, Abcam). It was experimentally shown that ab32049 antibody (Abcam) does not react with wild-type p53 in A549 cells. Binding of p53 to RPA in the parental or the p21^Waf1^-depleted cells was analyzed by western blotting following p53 immunoprecipitation with anti-p53 antibody (Pab240, Abcam). (**B**) Parental A549 cells or cells expressing p53 (His175) or p53 (His273) were synchronized with nocodazole, pulse treated with CPT at the entry to S phase and later maintained in drug-free medium. Bars represent the relative cell frequencies at different stages of the cell cycle at the indicated times. (**C)** HR frequency within pDR-GFP recombination substrate were measured 28 hours following CPT treatment.

We employed expression vectors for two p53 missense mutations found in human tumors - the conformational mutant p53-His175 and the DNA contact mutant p53-His273. We investigated whether in A549 cells co-expression of p53(His175) and p53(His273) that are known to functionally impair endogenous wild-type p53, affect RPA2 phosphorylation, stability of the p53/RPA complex and HR under CPT-induced replication stress. Both mutants were expressed in A549 cells to similar levels as detected with anti-p53 antibody (ab32049, Abcam) (Figure 5A). The antibodies that were developed to the p53 epitope corresponding to amino acids 374–393 do not react with wild-type p53 in A549 cells. As expected, p53 mutants abolish transcriptional activation of p21^Waf1^ as well as the inhibitory effect of p53 on RPA2 phosphorylation. In line with this result, RPA is released from the complex with p53 in the cells expressing either mutant. Both mutants upregulate HR in nocodazole-synchronized cells proceeding through S phase (Figure 5B, 5C).

## DISCUSSION

The importance of p53/RPA2 binding and the role of RPA2 phosphorylation in HR control by p53 was suggested by several studies [28, 29, 31]. Our findings confirm and extend these observations by showing that phosphorylation of RPA2 and therefore, the stability of the p53/RPA complex is regulated by p53 through transcriptional activation of its downstream target, p21^Waf1^. Predictably, changes in stability of the p53/RPA complex are accompanied by changes in HR rates.

In p53-proficient cells, we observed delays of RPA2 phosphorylation at Ser^29^, Tyr^21^ and Ser^4/8^ that correspond to CDK2, ATR/ATM, and DNA-PK consensus sites. As a potent inhibitor of CDKs, an activated p53 probably exerts its effect initially through inhibition of RPA2 phosphorylation at Ser^29^, which is followed by a delay in phosphorylation of other sites. Consistently, it was earlier shown that CPT-induced phosphorylation of CDK2 sites at Ser^23^ and Ser^29^ is required for subsequent RPA2 phosphorylation by ATR/ATM and DNA-PK at residues Thr^21^, Ser^33^ and Ser^4/8^. The sequential RPA2 phosphorylation is required for efficient HR DNA repair in response to replication arrest [9, 12, 33].

An investigation of p53 involvement in HR under conditions of genotoxic stress is complicated by its roles in regulation of the cell cycle and apoptosis. Probably due to the deficiency in DNA double-strand end resection in the G1 stage of cell cycle, DNA repair by HR is restricted to S and G2 stages. Therefore, under genotoxic stress, cell accumulation in G1 was suggested to account for the inhibitory effect of p53 on HR in many experiments [43]. To alleviate a potential contribution of cell cycle on HR assessment, we used synchronized cells that proceed through the G2-S stages without repopulating G1. S phase in both A549 and U2OS cells is significantly delayed in response to CPT, pointing to activation of an S phase checkpoint. Moreover, p53-proficient cells proceed through S phase with a similar pace to those deficient in p53 and p21^Waf1^. Others showed that p53 status does not affect cell cycle progression in similar settings [36]. This result is probably expected as p53 is not involved in regulation of the S phase checkpoint. On the other hand, phosphorylation of Thr^21^ and Ser^4/8^ is diminished in parental cells compared to derivatives deficient in p53 and p21^Waf1^. This may reflect the activation status of ATR, ATM and DNA-PK that regulate the S phase checkpoint in a CHK1- and CHK2-mediated fashion. Although a correlation between ATR/ATM and DNA-PK-dependent S phase checkpoint activation and RPA2 phosphorylation was observed in other studies [44], these events could be uncoupled [14, 45]. Thus, we suggest that the status of RPA2 phosphorylation is not indicative of S phase checkpoint activity, but rather reflects the intensity of DNA repair. Consistently, a faster removal of double-strand breaks, as judged by γ-H2AX immunostaining, was observed in p53- and p21^waf1^-depleted cells than in intact ones.

We previously reported that conformational and DNA contact mutants of p53, His175 and His273 [28], efficiently bound non-phosphorylated RPA. Nonetheless, both mutants failed to suppress spontaneous HR, suggesting that binding of RPA is necessary but not sufficient for inhibition of HR by p53. Inability of both mutants to bind recombination intermediates [46] was suggested to constitute an additional requirement for HR inhibition by p53. Here, we show that co-expression of the mutants and endogenous wild-type p53 in A549 leads to inhibition of p21^Waf1^, RPA2 hyperphosphorylation, dissociation of the p53/RPA complex and upregulation of HR. The inability of both mutants to bind recombination intermediates [46] probably contributes to the observed effect.

The mechanism of HR inhibition by the p53/RPA complex is unknown. It is plausible that RPA recruitment to ssDNA and its progressive phosphorylation would release p53. Phosphorylated forms of RPA acquire an affinity to Rad51 that closely collaborates with RPA in a search for homology and strand pairing at the initial stages of HR. Here, RPA brings p53 in close contact with recombination intermediates and Rad51. p53 is known to bind recombination intermediates and Holliday junctions *in vitro* showing preferences for heteroduplexes that contain nucleotide mismatches. Due to its intrinsic 3′-5′ exonuclease activity, p53 eliminates mispaired nucleotides, thus preventing error-prone homologous strand exchanges [18, 23, 47]. Such “proofreading” is accompanied by inhibition of the bacterial analog of Rad51 (reviewed in [48]). The combined evidence suggests that p53 may collaborate with RPA and Rad51 at the initial stages of recombination processing, probably by direct involvement in proofreading and eliminating errors that slow down the recombination process.

HR is considered to be one of the most accurate DNA repair mechanisms. One would expect that upregulated HR in p53-deficient cells would contribute to genome stability rather than destabilization. In reality, p53-depleted cells lacking an extended time for DNA repair due to a deficiency in G2 arrest rely on upregulted HR to survive DNA replication stress. The survived p53-negative cells are also deficient in p53 HR “proofreading” activity, and the p53 regulatory effect on nucleotide- and base-excision repairs (reviewed in [48]). Such cells would accumulate multiple genomic rearrangements leading to genomic instability. The outcome of CPT therapy is only a specific manifestation of the general phenomenon. Upregulated HR in the CPT-treated p53/p21^Waf1^-depleted cells contributes to survival and the propagation of cells carrying various types of DNA damage. In fact, low concentrations of the Top II inhibitor, etoposide, lead to a senescence-like G2 arrest of the p53-positive cells, and to continued proliferation and clonal expansion of the p53-deficient ones in a manner that is crucially dependent on ongoing HR [5]. Thus, our findings of the mechanisms underlying the regulation of HR contribute to an understanding of the genomic instability and drug resistance to CPT therapy in the cells with functionally depleted p53.

## MATERIALS AND METHODS

### Cell lines and plasmid constructs

We used human alveolar basal epithelial p53 wild-type (wt) positive cell line A549, human p53-negative non-small cell lung carcinoma cell line H1299, and human p53-positive osteosarcoma epithelial cell line U2OS. All cell lines were obtained from the American Type Culture Collection (ATCC) and were maintained in Dulbecco’s modified Eagle’s minimum essential medium (DMEM) supplemented with 10% fetal calf serum (Sigma Aldrich). A panel of p53 plasmid expression vectors was employed. CMV-based human p53(22.23), pCMV-neo-based, human p53(His173) and p53(His273) mutants were stably transfected into A549 cells using Lipofectamine^®^ 2000 Reagent (Thermo Fisher Scientific) according to the standard manufacturer’s protocol.

### Cell synchronization, CPT treatment, and viability testing

The parental A549 cell line or its derivatives were synchronized at G2/M by treatment with nocodazole (Sigma-Aldrich) at a concentration 40 ng/ml for 26 hours. U2OS cells were synchronized by nocodazole treatment for 12 hours at a final concentration 50 ng/ml, respectively. Cells were washed three times with warm medium and then maintained in drug-free medium. To assess cell cycle progression, the cells were analyzed by flow cytometry at different time intervals. At the entrance to S phase, 19 or 10 hours following nocodazole removal, A549 or U2OS cells were treated with 500 nM CPT (Sigma-Aldrich) for 1 hour, washed 2 times with warm medium and then maintained in drug-free medium. Cells were harvested at different time intervals, stained with propidium iodine and analyzed by flow cytometry for cell cycle distribution on a FACSCalibur analyzer (BD Biosciences). Cell viability (apoptosis) was detected using Annexin V-FITC Apoptosis Detection Kit (Abcam) according to the manufacturer’s protocol.

### Homologous recombination assay

Single-cell-derived H1299 subclones carrying chromosomally-integrated pDR-green fluorescent protein (GFP), pDR-GFP [49] were selected and screened for their proficiency to undergo HR in response to replication arrest induced by CPT. A representative subclone was selected and used in subsequent experiments. The analysis of HR in response to replication arrest induced by CPT was performed as described previously [41]. The experiments were performed in triplicate. Briefly, parental cells or derivatives of A549 or U2OS cells were synchronized and treated with CPT at the entrance to S phase as described above. The cells were subsequently washed in warm medium and then incubated in drug-free medium. Twenty seven or twenty two hours later, A549 and U2OS cells were collected and subjected to flow cytometric analysis for GFP-positive cells. For each analysis, at least 5 × 10^5^ cells were processed. Non-parametric Chi-Square tests were used to validate the differences in GFP expression between the samples. Data were analyzed using STATISTICA (StatSoft, Inc., Tulsa OK).

### p53, p21^waf1^ silencing, RPA2 silencing and replacement strategy

Validated siRNAs developed to the coding region of human p53 (GGUUUUUACUGUGAGGGAUTT) and CDKN1A gene encoding p21^waf1^ (GGCCCGCUCUACAUCUUCUTT) were obtained from Thermo Fisher Scientific. Replacement of endogenous RPA2 with the recombinant Myc-tagged human wild-type RPA2 or its phosphorylation-deficient and phosphorylation-mimetic mutants, RPA2A and RPAD4, respectively, was described earlier [33]. Briefly, retrovirally-infected A549 clones were grown for 48 hours in medium lacking doxycycline to allow ectopic RPA2 expression. Endogenous RPA2 was then down-regulated using an siRNA (top strand sequence, 5′-AAC CUA GUU UCA CAA UCU GUU-3′) targeting the 3′-untranslated region of the RPA2 mRNA. Silencing was achieved using Stealt^™^/siRNA Transfection Protocol using Lipofectamine^R^ 2000 (Thermo Fisher Scientific) per the manufacturer’s instructions. Representative levels of recombinant RPA2 were analyzed by western blot.

### [H^3^]*-*thymidine incorporation assay

Cells were labeled with ^3^H-Thymidine, at 0.1 µCi/100 µl for 1 h. After labeling, the cells were washed twice with PBS and precipitated with 200 µl of TCA (Sigma-Aldrich) for 30 minutes at room temperature. TCA was aspirated and the precipitate was lysed in 50 µl of 1 M NaOH for another 30 minutes and neutralized with 40 µl of 1 M HCl. The incorporated ^3^H-Thymidine was counted in a MicroBeta Scintillator (PerkinElmer).

### Immunoprecipitation, western blotting, immunofluorescence and antibodies

Immunoprecipitation were performed in buffer containing 10 mM Tris HCl, pH 7.4; 20 mM NaCl; 0.5 mM EDTA; 0.5 mM EGTA; 0.01% NP40; 10 mg/ml aprotinin (Sigma-Aldrich); 10 mg/ml leupeptin (Sigma-Aldrich) and 500 μM PMSF (Sigma-Aldrich). For western blot analysis, cells were directly lysed in SDS-PAGE sample buffer, and the lysates were separated by SDS-PAGE. Proteins were immobilized onto Protran nitrocellulose membranes (0.2-μm pore size). We used polyclonal anti-p53 antibody (Pb240, Abcam), monoclonal anti-mutant p53 (ab32509, Abcam), polyclonal anti-CDKN1 (p21Waf1) (Pharmingen), polyclonal anti-phospho-H2AX (γ-H2AX) (Thermo Fisher Scientific), monoclonal anti-RPA1 (Oncogene Science), monoclonal anti-RPA2 ([9H8], Neomarkers), monoclonal anti-c-Myc (Bethyl Biolabs), polyclonal anti-RPA2 (NeoMarkers), anti-RPA2 Thr(P)^21^ (Abcam), and anti-RPA2 Ser(P)^4^/Ser(P)^8^ (Bethyl Laboratories). Custom anti-RPA2Ser(P)^29^ antibodies were produced by (Bethyl Laboratories) as described in [33]. Goat anti-rabbit or anti-mouse IgG conjugated with horseradish peroxidase (Amersham) were used as secondary antibodies. For developing western blots, western wash buffer (PBS containing Tween 20 (0.3%, v/v), 5 mM sodium fluoride, and 0.1 mM sodium orthovanadate) was used. All phospho-specific antibodies were incubated in western wash buffer containing nonfat dry milk (0.5%, w/v) and bovine serum albumin (0.5%, w/v). The secondary antibodies and non-phospho-specific primary antibodies were incubated in western wash buffer containing 0.2% (w/v) nonfat dry milk. Detection was carried out using enhanced chemiluminescence (Amersham Biosciences).

For immunofluorescence we used primary mouse monoclonal Anti-phospho-Histone H2A.X (Ser139) antibody (Millipore Sigma, Cat# 05–636) and secondary Texas Red-labeled Goat anti-mouse IgG (H+L) polyclonal antibody (Themofisher Scientific, Cat# T-862). Staining was performed on methanol-fixed cells grown on 4-well Lab-Tek II glass slides (Nunc) according to standard protocol. Nuclei were counterstained with DAPI (Vector Laboratories). Images were captured on an Olympus AX70 microscope with a 100× objective using a RT3 camera (Diagnostic Instruments) and analyzed using Spot software v 5.2 (Diagnostic Instruments).
